# Impact of the COVID-19 pandemic on individuals with nystagmus and an exploration of public assumptions about the condition: an electronic questionnaire study

**DOI:** 10.1186/s12886-022-02484-x

**Published:** 2022-06-20

**Authors:** Katherine Rennie, Rajeeth Alagendran, Helena Lee, Helen Griffiths, Nystagmus U. K. Eye Research Group (NUKE), M Theodorou, H Lee, J Self, F Shawkat, P Carter, J Erichsen, M Dunn, L McIlreavy, N Thomas, K Ward, J Whittle, J Sanders, C Harris, R McLean, D Lawrence, S Ricketts, A Gliksohn, H Griffiths, M Thomas, H Kuht, H Kubavat, M Woodhouse, G Arblaster, James Self

**Affiliations:** 1 Clinical and Experimental Sciences, Faculty of Medicine, University of Southampton, Southampton, SO16 6YD UK; 2 Sheffield Children’s Hospital, Sheffield, Yorkshire UK

**Keywords:** Nystagmus, COVID-19, Survey, Public awareness

## Abstract

**Purpose:**

Nystagmus is a disorder characterized by uncontrolled, rhythmic oscillations of the eyes. It often causes reduced visual function beyond reduced visual acuity alone. There is a paucity of literature regarding the public understanding of nystagmus, and there are no published data on the impact of the COVID-19 pandemic on people living with the condition. This study explores the self-reported impact of the COVID-19 pandemic on those with nystagmus, and examines both public understanding of how nystagmus affects people who have it and the perceptions of public understanding by those with the condition and their carers.

**Methods:**

A qualitative questionnaire was designed following a stakeholder engagement process. This questionnaire was advertised via social media platforms and charity websites to achieve widespread recruitment. Data were collected between November and December 2020. Participants were divided into two groups based on their response to the question: “Do you, or anyone you know well, have nystagmus?”. Questions were posed to participants in a purpose-built, branching survey. The resulting data were analyzed using descriptive and inferential statistical methods.

**Results:**

One thousand six hundred forty-five respondents were recruited, of which 849 (51.6%) answered “Yes” to the initial filtering question. Analysis showed that, broadly, public understanding of nystagmus differs from the perception of it by those with nystagmus and their carers, that the COVID-19 pandemic has had a significant impact on those with nystagmus, and that respondents who have met someone with nystagmus, even briefly, tend to have a greater understanding of the impact of the condition.

**Conclusion:**

This study highlights the lack of public awareness regarding nystagmus and suggests opportunities to increase the awareness of nystagmus without the need for extensive knowledge of the condition. The COVID-19 pandemic has posed additional difficulties for those living with nystagmus, which is likely to be comparable among those with similar ocular disorders.

**Supplementary Information:**

The online version contains supplementary material available at 10.1186/s12886-022-02484-x.

## Introduction

Nystagmus is a disorder of eye movement, characterized by an uncontrolled, rhythmic oscillation of the eyes [1]. It is associated with a variety of underlying causes and results in significant visual loss. Individuals with nystagmus will often have limited or reduced visual acuity, and there are often associated visual symptoms related to the underlying cause, including significant retinal dysfunction, photosensitivity, or photophobia [2]. In the UK, nystagmus is estimated to affect 24 per 10,000 people, and treatment options for the majority of patients are currently limited [3].

It has been shown that individuals with nystagmus are more likely to have reduced social functioning [4, 5]. The lack of public understanding regarding nystagmus has also been shown to have negative impacts on those living with the condition [6]. There are no published data on the impact of the COVID-19 pandemic on people with nystagmus. One study on eye disorders more broadly showed that participants felt that their eye conditions had caused increased difficulties in coping during the lockdowns [7]. The present study explores the self-reported impact of the COVID-19 pandemic on those living with nystagmus and analyses both public understanding of how nystagmus affects people who have it and the perceptions about public understanding by those with the condition and their carers.

## Materials and methods

This study had ethical approval (East Midlands-Leicester South Research Ethics Committee 16/EM/0418) and was conducted in accordance with the Helsinki declaration. Prior to designing the questionnaire, patient and public involvement work was undertaken. This was in the form of feedback and patient anecdotes that were gathered from those who had nystagmus and their carers. These excerpts were collected from numerous sources, including the charity Nystagmus Network and consultants working in ophthalmology, and included information about some of the challenges that individuals with nystagmus faced as well as suggestions for possible questions. These were grouped into categories of recurring themes, from which questions were formed to create the questionnaire based on the most common areas of concern and designed to yield maximum relevant data whilst keeping the survey as brief as possible and in accordance with the needs of some visually impaired respondents. The questionnaire was created on Microsoft Forms and contained a video of nystagmus to ensure that members of the public were clear about the nature of the condition. This pilot questionnaire was tested by consultants, research members involved in the project, medical students, and laypeople, in addition to those with nystagmus and their carers, to identify any technical or grammar issues that needed to be resolved.

After the appropriate amendments were made, the Microsoft Forms link was posted to various social media platforms including Facebook, Twitter and WhatsApp as well as Nystagmus Network and other charity websites. The final questionnaire can be viewed in appendix 1. The questionnaire was also promoted after the researchers gave talks in certain locations. Participants who completed the questionnaire were then asked to share the survey with other individuals as broadly as possible in order to reach as many people who had never heard of the condition as possible, in addition to those with nystagmus and their carers.

Inclusion criteria were anyone completing the entire survey, and as it was not possible to have an incomplete survey response, there were no exclusion criteria. Responses were anonymous with the only identifying question being a single question about whether respondents were UK-based or not.

One thousand six hundred forty-five participants were recruited within the study window of 6th November 2020—10^th^ December 2020.

The participant responses were collated in an Excel spreadsheet. The data were manipulated from the Excel spreadsheet to allow input into SPSS version 27, for analysis.

## Results

The population included in the study was split into two groups, with a nystagmus group (n = 849) and a public group (n = 796). The nystagmus group comprised people with the condition and people who were very close to someone with nystagmus, for example, a parent or carer. The public group was subdivided into those who did not know anyone with nystagmus (n = 516) and those who did not have nystagmus but had met someone with the condition (n = 280).

### What proportion of people in the nystagmus group reported facing additional challenges during the COVID-19 pandemic?

Of the respondents who either had nystagmus or knew someone with nystagmus well, 40.5% said those with nystagmus experienced additional challenges due to the COVID-19 pandemic. Regarding specific challenges, 51.1% of those with nystagmus experienced difficulties with conducting video meetings, and 75.5% faced challenges with social interactions.

Table 1 displays the number and percentage of respondents who self-reported additional challenges due to their nystagmus, as well as the number and percentage of the public who anticipated such challenges for those with nystagmus. A significant proportion of the public perceived that nystagmus creates additional challenges regarding social interactions and conducting video meetings, with proportions of 89.7% and 87.8%, respectively. A significantly higher percentage of the public group thought that issues regarding COVID-19, social interaction, and video meetings would affect people with nystagmus than members of the nystagmus group who said that this issue has affected them.

**Table 1:** Table comparing responses in the nystagmus group against those in the public group, regarding if challenges had been faced (or would be expected) with social interaction, COVID-19, or video meetings

	Public group **(*n* = 796)**	Nystagmus group **(*n* = 849)**
	Number of “Yes” responses (%)	Number of “Yes” responses (%)
COVID-19	528 (66.3)	344 (40.5)
Social Interaction	714 (89.7)	641 (75.5)
Video meetings	699 (87.8)	434 (51.1)

Table 2 highlights the variation in responses of members of the public asked to estimate what proportion of individuals with nystagmus were affected. Approximately one-third of the public sample (35.1%) predicted that 51–75% of individuals with nystagmus faced challenges conducting video meetings in the pandemic. Regarding individuals with nystagmus, 51.5% said they were affected. There are notable proportions of the public that potentially underestimated the impact of nystagmus during the pandemic, with 33.7% believing there is no impact and 6.3% perceiving that low proportions of individuals with the condition are affected (1–25% of people with nystagmus). Regarding social interaction, the majority of the public group believed that 76–100% of individuals with nystagmus would be affected. This is notable as 75.5% of those from the nystagmus group felt that they were affected.

**Table 2:** Table displaying responses from the public group regarding perceived estimates of the number of people with nystagmus affected by each aspect, compared to actual numbers affected in the nystagmus group

	Number of responses from public group **(*n* = 796)** (%)	Actual Number affected from Nystagmus Group **(*n* = 849)** (%)
COVID-19		344 (40.5)
0%	268 (33.7)	
1–25%	50 (6.3)	
26–50%	139 (17.5)	
51–75%	199 (25)	
76–100%	140 (17.6)	
Social Interaction		641 (75.5)
0%	82 (10.3)	
1–25%	48 (6)	
26–50%	176 (22.1)	
51–75%	263 (33)	
76–100%	227 (28.5)	
Video meetings		434 (51.1)
0%	97 (12.2)	
1–25%	48 (6)	
26–50%	161 (20.2)	
51–75%	279 (35.1)	
76–100%	211 (26.5)	

### Can people in the nystagmus group accurately predict the public’s assumptions of the condition?

Table 3 shows that, for all aspects, a lower proportion of participants with nystagmus perceived that the public thought the condition affected each lifestyle aspect than the true value. For example, only 39.8% of participants with nystagmus assumed the public would suggest that children with the condition would need more support at school, whereas 81.0% of the public anticipated this. In addition, for day vision variance, 46.4% of participants with nystagmus assumed the public would suggest that those with nystagmus face difficulties with day vision variance. This is notable as 83.8% of the public anticipated this.

**Table 3:** Table comparing responses between nystagmus group and public group

	Public group **(*n* = 796)**	Nystagmus group **(*n* = 849)**
	Number of “Yes” responses (%)	Number of “Yes” responses (%)
Visual Impairments	556 (69.8)	507 (59.7)
Child School Support	645 (81.0)	338 (39.8)
Driving License	546 (68.6)	418 (49.2)
Day Vision Variance	667 (83.8)	394 (46.4)
Smartphone Usage	683 (85.8)	629 (74.1)

Those in the nystagmus group were asked to predict how they thought the public would respond regarding difficulties faced with each aspect. Day vision variance is whether the person’s vision changes throughout the day (i.e. if it improves or gets worse as the day progresses)

### Does the public understanding of nystagmus depend on the person’s previous exposure to the condition?

Members of the public who had met someone with nystagmus tended to believe that significantly higher proportions of individuals with the condition were affected by each aspect (video meetings, child school support, driving license and smartphone usage aspects) compared to those who had never met someone with nystagmus. This can be seen in Table 4, as responses from the “public met” group are clustered around the higher percentage ranges, whilst responses from the “public had not met” group skewed more towards the lower percentage ranges, including 0%. The option 0% indicates “no”, showing that participants did not believe that nystagmus affects the stated aspect. An exception to this is driving license qualification, where 13.9% of the public who had met someone with nystagmus thought 76–100% would not be able to obtain a driving license. whereas in the public that had not met someone with nystagmus this figure was 22.7%. Notably, 17.4% of people who had not met someone with nystagmus thought that smartphone usage would not be affected by nystagmus, however this figure was 8.2% in those who had met a person with nystagmus.

**Table 4:** Table comparing ranged percentage responses between those that had met someone with nystagmus and those that had not

	Public Met **(*n* = 280)**	Public Had Not Met **(*n* = 516)**
	Number of Responses (%)	Number of Responses (%)
COVID-19		
0%	84 (30)	184 (35.7)
1–25%	14 (5)	36 (7)
26–50%	48 (17.1)	91 (17.6)
51–75%	80 (28.6)	119 (23.1)
76–100%	54 (19.3)	86 (16.7)
Social Interaction		
0%	20 (7.1)	62 (12)
1–25%	18 (6.4)	30 (5.8)
26–50%	56 (20)	120 (23.3)
51–75%	103 (36.8)	160 (31)
76–100%	83 (29.6)	144 (27.9)
Video meetings		
0%	25 (8.9)	72 (14)
1–25%	13 (4.6)	35 (6.8)
26–50%	48 (17.1)	113 (21.9)
51–75%	119 (42.5)	160 (31)
76–100%	75 (26.8)	136 (26.4)
Visual Impairments		
0%	68 (24.3)	172 (33.3)
1–25%	19 (6.8)	28 (5.4)
26–50%	55 (19.6)	81 (15.7)
51–75%	70 (25)	130 (25.2)
76–100%	68 (24.3)	105 (20.3)
Child School Support		
0%	40 (14.3)	111 (21.5)
1–25%	22 (7.9)	48 (9.3)
26–50%	65 (23.2)	114 (22.1)
51–75%	101 (36.1)	134 (26)
76–100%	52 (18.6)	109 (21.1)
Driving License		
0%	89 (31.8)	161 (31.2)
1–25%	35 (12.5)	33 (6.4)
26–50%	54 (19.3)	88 (17.1)
51–75%	63 (22.5)	117 (22.7)
76–100%	39 (13.9)	117 (22.7)
Day Vision Variance		
0%	41 (14.6)	88 (17.1)
1–25%	17 (6.1)	30 (5.8)
26–50%	70 (25)	129 (25)
51–75%	89 (31.8)	162 (31.4)
76–100%	63 (22.5)	107 (20.7)
Smartphone Usage		
0%	23 (8.2)	90 (17.4)
1–25%	11 (3.9)	28 (5.4)
26–50%	46 (16.4)	71 (13.8)
51–75%	87 (31.1)	154 (29.8)
76–100%	113 (40.4)	173 (33.5)

0% indicates the answer "no effect", indicating the participants who believed that nystagmus does not affect a certain aspect

Table 5 compares the ranged percentage responses between those who had met someone with nystagmus and those who have never heard of nor met someone with nystagmus. These data exhibit a similar trend to that of Table 4, with the differences being more pronounced. The skew of responses towards the lower percentage ranges is even greater in the “public had never heard nor net” group in comparison to the “public had met” group. Regarding visual impairment, 38.9% of participants who had never heard of nor met anyone with nystagmus believed the condition did not cause visual impairments, compared to the lower figure of 24.3% of participants who had met someone with nystagmus.

**Table 5:** Table comparing the ranged percentage responses between those that had met someone with nystagmus and those that have never heard of nor met someone with nystagmus

	Public Met **(*n* = 280)**	Public Had Never Heard nor Met **(*n* = 332)**
	Number of Responses (%)	Number of Responses (%)
COVID-19		
0%	84 (30)	120 (36.1)
1–25%	14 (5)	26 (7.8)
26–50%	48 (17.1)	54 (16.3)
51–75%	80 (28.6)	83 (25)
76–100%	54 (19.3)	49 (14.8)
Social Interaction		
0%	20 (7.1)	45 (13.6)
1–25%	18 (6.4)	22 (6.6)
26–50%	56 (20)	73 (22)
51–75%	103 (36.8)	101 (30.4)
76–100%	83 (29.6)	91 (27.4)
Video meetings		
0%	25 (8.9)	51 (15.4)
1–25%	13 (4.6)	17 (5.1)
26–50%	48 (17.1)	69 (20.8)
51–75%	119 (42.5)	101 (30.4)
76–100%	75 (26.8)	94 (28.3)
Visual Impairments		
0%	68 (24.3)	129 (38.9)
1–25%	19 (6.8)	17 (5.1)
26–50%	55 (19.6)	46 (13.9)
51–75%	70 (25)	85 (25.6)
76–100%	68 (24.3)	55 (16.6)
Child School Support		
0%	40 (14.3)	78 (23.5)
1–25%	22 (7.9)	30 (9)
26–50%	65 (23.2)	68 (20.5)
51–75%	101 (36.1)	84 (25.3)
76–100%	52 (18.6)	72 (21.7)
Driving License		
0%	89 (31.8)	112 (33.7)
1–25%	35 (12.5)	15 (4.5)
26–50%	54 (19.3)	55 (16.6)
51–75%	63 (22.5)	69 (20.8)
76–100%	39 (13.9)	81 (24.4)
Day Vision Variance		
0%	41 (14.6)	63 (19)
1–25%	17 (6.1)	20 (6)
26–50%	70 (25)	82 (24.7)
51–75%	89 (31.8)	106 (31.9)
76–100%	63 (22.5)	61 (18.4)
Smartphone Usage		
0%	23 (8.2)	61 (18.4)
1–25%	11 (3.9)	21 (6.3)
26–50%	46 (16.4)	48 (14.5)
51–75%	87 (31.1)	105 (31.6)
76–100%	113 (40.4)	97 (29.2)

## Discussion

A large sample size of respondents was recruited for this study (n = 1645), making this report the largest questionnaire study on nystagmus as far as we are aware. Overall, this study shows that people with nystagmus have been negatively impacted by the COVID-19 pandemic, particularly with regards to conducting video meetings and socially interacting with others. Most individuals who faced challenges have struggled with social interaction and with working from home, which may have involved the use of new technology and the use of video calls. As symptoms of nystagmus vary between individuals, and the previous experience of using technology can also vary, this could explain why some individuals have faced greater challenges than others.

It is important to note that the ages of participants were not collected during this study, so it not possible to look at the demographics of those who have answered the survey.

Further to this, results from Tables 1 and 2 show that the public either overestimated or accurately estimated the impact that nystagmus has on social interactions, dealing with the COVID-19 pandemic, and video conferencing. This indicates that the majority of the public appear to be able to anticipate how the condition would impact individuals during a lockdown, despite only a brief introduction video showing the eye movement abnormality. These additional challenges faced by individuals with nystagmus during the COVID-19 pandemic could have had many differing root causes. McLean et al. published a paper regarding living with nystagmus and stated that not fitting in and cosmetic appearance of nystagmus were two of six domains that were adversely affected by nystagmus [8]. In the case of COVID-19, where much more has moved online, these issues could be more profound and obvious on a computer screen, leading individuals with nystagmus to struggle more.

It is plausible that the inclusion of questions describing specific tasks prompted respondents to make assumptions regarding individuals with nystagmus based on their prior experience of the condition or their assumptions about the visual potential for people with nystagmus as a group. The public group was heterogeneous and skewed, consisting of 280 participants who had met someone with nystagmus and 516 individuals who had not. This could explain the wide range of responses to the question asking whether individuals with nystagmus faced extra challenges during the COVID-19 pandemic, where large proportions inaccurately stated that individuals with nystagmus would face either few or no additional challenges. This could therefore be a specific aspect to highlight when trying to educate and increase awareness of nystagmus amongst members of the public.

Concerning perceptions of public beliefs by people with nystagmus, it can be argued that individuals with nystagmus inaccurately predicted the public’s assumptions of their condition, with the majority believing that the public would not understand the difficulties that they face. This discordance is strikingly highlighted in Table 3 when looking at data for child school support.

It must be considered that the use of mean percentages from ranged data may not allow for accurate comparisons to be made, as means calculated from ranged data are only estimates. When calculating estimated means, the middle value is assumed to be the choice participants intend, from any ranged data options they select. However, this may not be the choice participants intended to make.

The conclusion can be drawn that people with nystagmus tended to predict that the public would not think that the condition affects their daily lifestyle as much as it actually does. This may result in a negative feeling amongst those with nystagmus that the public does not understand the condition or appreciate the full impact it has on daily life.

A key conclusion drawn from this study is that members of the public had a greater understanding of challenges faced by individuals with nystagmus if they had met someone with nystagmus, however briefly, or if they had even heard of the condition. This suggests that greater public awareness of the condition could be achieved through increased exposure of the condition through various media and online methods of promotion without the need for detailed education programs. For example, the awareness of nystagmus could be achieved anecdotally, with a focus on different people with nystagmus talking about their condition as opposed to clinicians explaining it. This could be achieved through a series of online videos or live teaching sessions in schools, where particular attention can be raised with regards to the variance in visual impairment and how this affects individuals with the condition differently. It could perhaps also be achieved by people speaking openly about their nystagmus on public platforms.

From this study, it is not possible to say if people with nystagmus struggled with other aspects of the pandemic or faced additional challenges (for example socially isolating or shopping during a lockdown). Further qualitative questionnaire studies regarding nystagmus could be carried out to identify specific issues that individuals with nystagmus have faced during a lockdown and whether these are common to other disorders of vision.

### Strengths and limitations

Although conventional surveys usually include three options, the omission of a “don’t know” option and the choice to solely include “yes” and “no” responses in this survey allowed for the aims to be reached and appropriate analysis to be completed. In addition, it pushes respondents to make a decision as they would do in the “real-world” when meeting someone with nystagmus [9, 10].

A limitation of this study was that there was the risk of participants in the study only being from the social groups of the researchers, which is a known limitation of online survey research [11]. This was mitigated by encouraging participants to share the questionnaire with other individuals to reach a broader range of people who both knew and had never heard of nystagmus. This allowed for rapid recruitment of additional participants and an overall larger sample size to be attained [10, 11].

A limitation is the selection of a non-validated survey tool for the questionnaire. However, this was chosen due to wanting to get the information out quickly as this information is important to those living with nystagmus, and it is key to disseminate the information to facilitate and educate during the COVID-19 pandemic.

## Supplementary Information

**Additional file 1.**

## Data Availability

The datasets used and/or analyzed during the current study are available from the corresponding author on reasonable request.
