# Supplementary material for: Impact of the COVID-19 pandemic on individuals with nystagmus and an exploration of public assumptions about the condition: an electronic questionnaire study
Source: BMC Ophthalmol. 2022 Jun 20;22:268. doi: 10.1186/s12886-022-02484-x (PMC9206891; doi:10.1186/s12886-022-02484-x)
Supplement: Supplementary file 1 — Additional file 1. [file 12886_2022_2484_MOESM1_ESM.docx]

**Appendix 1 – The questionnaire**

The questionnaire comprised of 5 sets of questions. Those in the nystagmus group answered questions from sets 1-3 whilst those in the public group answered questions from sets 1,4 and 5. Certain questions were only shown to respondents who answered “Yes” to the previous question (i.e. questions asking for ranged percentage response). Thus, the total number of questions answered varied between participants based on the responses given.

The following flow chart shows the sequences of sets asked:

No

Yes

The questions in the sets were as follows:

Figure 1 Flow chart depicting which sets of questions participants would be asked to answer

**Set 1:**

First, participants were shown a video of someone with nystagmus so that they knew what the condition looked like.
• **Are you a UK resident?** (Yes/No)
• **Have you heard of the word “Nystagmus” before this survey?** (Yes/No)

• **Have you ever met anyone with nystagmus?** (Yes/No)

- Those that answered “No” to this question were shown questions from set 4
  and 5 to answer.
- Those that answered “Yes” to this question continued with set 1.

• **Do you, or anyone you know well, have nystagmus?** (Yes/No)

- Those that answered “Yes” to this question were shown questions from set 2 and 3 to answer.
- Those that answered “No” to this question were shown questions from set 4 and 5 to answer.

**Set 2:**• **Has nystagmus caused challenges with social interactions for the person you know with nystagmus (this might be you)?** (Yes/No)
• **Has the COVID-19 pandemic caused additional challenges for the person you know
with nystagmus, because of their nystagmus?** (Yes/No)
• **Do video meetings present more challenges for the person you know with
nystagmus, because of their nystagmus?** (Yes/No) **Set 3:**• **How do you think that someone who does not know anyone with nystagmus well, would answer this question: Do you think that nystagmus would cause visual impairment?** (Yes/No)

- Those that answered “Yes” to this question were shown the following question:

**How do you think that someone who does not know anyone with nystagmus well, would answer this question: What percentage of people with nystagmus would be visually impaired?** (1-25% / 26-50% / 51-75% / 76-100%)

• **How do you think that someone who does not know anyone with nystagmus well, would answer this question: Do you think that a child who can read and play ball games could require additional support in school because they have nystagmus?** (Yes/No)

- Those that answered “Yes” to this question were shown the following question:

**How do you think that someone who does not know anyone with nystagmus well, would answer this question: What percentage of children with nystagmus who can read and play ball games would require additional support in school?** (1-25% / 26-50% / 51-75% / 76-100%)

• **How do you think that someone who does not know anyone with nystagmus well, would answer this question: Could people with nystagmus qualify for a driving license?** (Yes/No)

- Those that answered “Yes” to this question were shown the following question:

**How do you think that someone who does not know anyone with nystagmus well, would answer this question: What percentage of people with nystagmus would qualify for a driving license?** (1-25% / 26-50% / 51-75% / 76-100%)

• **How do you think that someone who does not know anyone with nystagmus well, would answer this question: Could nystagmus cause varying vision during the day?** (Yes/No)

- Those that answered “Yes” to this question were shown the following question: **How do you think that someone who does not know anyone with nystagmus well, would answer this question: What percentage of people with nystagmus would experience varying vision during the day?** (1-25% / 26-50% / 51-75% / 76-100%)

• **How do you think that someone who does not know anyone with nystagmus well, would answer this question: If someone has poor vision and nystagmus, do you think they could use a smartphone?** (Yes/No)

- Those that answered “Yes” to this question were shown the following question: **How do you think that someone who does not know anyone with nystagmus well, would answer this question: What percentage of people with poor vision and nystagmus could use a smartphone?** (1-25% / 26-50% / 51-75% / 76-100%)

**Set 4:**• **Do you think that people with nystagmus might experience challenges with social
interactions due to their nystagmus?** (Yes/No)

- Those that answered “Yes” to this question were shown the following question: **What percentage of people with nystagmus do you think would have experienced challenges with social interactions due to their nystagmus?** (1-25% / 26-50% / 51-75% / 76-100%)

• **Do you think that people with nystagmus might have faced additional challenges
due to their nystagmus during the COVID-19 pandemic?** (Yes/No)

- Those that answered “Yes” to this question were shown the following question: **What percentage of people with nystagmus would you think have faced additional challenges due to their nystagmus during the COVID-19 pandemic?** (1-25% / 26-50% / 51-75% / 76-100%)

• **Do you think that people with nystagmus might experience additional challenges
with video meetings due to their nystagmus?** (Yes/No)

- Those that answered “Yes” to this question were shown the following question: **What percentage of people with nystagmus would you think experience additional challenges with video meetings due to their nystagmus?** (1-25% / 26-50% / 51-75% / 76-100%)

**Set 5:**

• **Do you think that people with nystagmus would be visually impaired?** (Yes/No)

- Those that answered “Yes” to this question were shown the following question: **What percentage of people with nystagmus would be visually impaired?** (1-25% / 26-50% / 51-75% / 76-100%)

• **Do you think that a child who can read and play ball games could require
additional support in school because they have nystagmus?** (Yes/No)

- Those that answered “Yes” to this question were shown the following question: **What percentage of children with nystagmus who can read and play ball games would require additional support in school?** (1-25% / 26-50% / 51-75% / 76-100%)

• **Could people with nystagmus qualify for a driving license?** (Yes/No)

- Those that answered “Yes” to this question were shown the following question: **What percentage of people with nystagmus would qualify for a driving license?** (1-25% / 26-50% / 51-75% / 76-100%)

• **Could nystagmus cause varying vision during the day?** (Yes/No)

- Those that answered “Yes” to this question were shown the following question: **What percentage of people with nystagmus would experience varying vision during the day?** (1-25% / 26-50% / 51-75% / 76-100%)

• **If someone has poor vision and nystagmus, do you think they could use a
smartphone?** (Yes/No)

- Those that answered “Yes” to this question were shown the following question: **What percentage of people with poor vision and nystagmus could use a smartphone?** (1-25% / 26-50% / 51-75% / 76-100%)
